# Easy One-Pot Decoration of Graphene Oxide Nanosheets by Green Silver Nanoparticles

**DOI:** 10.3390/ijms26020713

**Published:** 2025-01-16

**Authors:** Ileana Ielo, Federica De Gaetano, Elpida Piperopoulos, Giovanna De Luca, Sabrina Conoci

**Affiliations:** 1 Department of Chemical, Biological, Pharmaceutical and Environmental Sciences, University of Messina, Viale F. Stagno d’Alcontres 31, 98166 Messina, Italy; federica.degaetano@unime.it (F.D.G.); sabrina.conoci@unime.it (S.C.); 2 Technology and Research on Energy, Environment and Safety Materials, Department of Engineering, University of Messina, Contrada Di Dio, 98166 Messina, Italy; elpida.piperopoulos@unime.it; 3 Department of Chemistry “Giacomo Ciamician”, University of Bologna, Via Selmi 2, 40126 Bologna, Italy; 4 Institute for Microelectronics and Microsystems, National Research Council (CNR-IMM), Ottava Strada n.5, 95121 Catania, Italy

**Keywords:** silver nanoparticles, graphene oxide, one-pot nanoparticle synthesis, green nanoparticle synthesis, nanocomposite

## Abstract

In this study, we developed a facile one-pot synthesis of a nanocomposite consisting of silver nanoparticles (AgNPs) growing over graphene oxide (GO) nanoflakes (AgNPs@GO). The process consists of the in situ formation of AgNPs in the presence of GO nanosheets via the spontaneous decomposition of silver(I) acetylacetonate (Ag(acac)) after dissolution in water. This protocol is compared to an ex situ approach where AgNPs are added to a waterborne GO nanosheet suspension to account for any attractive interaction between preformed nanomaterials. The systems under investigation are characterized by UV/vis absorption spectroscopy, dynamic light scattering (DLS), zeta potential (Z-Pot), and scanning electron microscopy with energy-dispersive X-ray spectroscopy (SEM-EDX). The stability of the AgNPs@GO composite suspension is tested as a function of GO concentration (0–67 μg/mL) while maintaining a constant Ag content (14.4 μg/mL), exhibiting excellent stability over time up to an Ag-to-GO mass ratio of 0.58.

## 1. Introduction

The graphene manufacturing Industry, particularly focused on graphene nanomaterials, holds immense potential for significant economic impact worldwide. While it is still in its early stages of commercialization, the global graphene market is projected to reach a value of USD 200 million by 2026 [1]. The increasing need for advanced materials in various industries, including the development of antibacterial coatings [2] and quantum technology devices [3], is driving this growth. Among graphene-based nanocomposites, silver nanoparticles/graphene oxide nanosheets represent a promising class of nanoarchitectures with diverse applications in fields ranging from electrocatalysis [4,5,6,7] to biomedicine [8,9] or sensing [10,11]. The unique properties of silver nanoparticles (AgNPs) and graphene oxide (GO), including high surface area, electrical conductivity, tunable surface chemistry, and (bio)active properties, synergistically contribute to the enhanced performance and multifunctionality of the nanocomposite material [5,6,12,13,14]. Ensuring the chemical stability of such nanocomposites, e.g., their ability to maintain their structural integrity, prevent agglomeration, and resist chemical degradation under various environmental conditions, is paramount for their practical utilization, processability, and long-term efficacy [15]. In this context, finding simple routes for fabricating Ag-decorated GO nanosheets and investigating the chemical stability of their suspensions is essential for elucidating the behavior in real-world applications and guiding the development of robust and reliable nanomaterials.

In our study, the synthesis of the AgNPs/GO nanocomposite (AgNPs@GO) is attained using an in situ approach which consists of forming AgNPs from an Ag^+^ precursor in the presence of suspended GO nanosheets. These results are then compared to the ex situ protocol, where preformed AgNPs are combined with GO. The synthesized AgNPs and AgNPs@GO composites underwent comprehensive characterization using UV/Vis absorption spectroscopy, dynamic light scattering (DLS), zeta potential analysis, scanning electron microscopy (SEM), and energy-dispersive X-ray (EDX) spectroscopy. Our straightforward, environmentally friendly, and reproducible synthesis advances nanocomposite production by providing valuable insights into their structural and colloidal properties. This knowledge is essential to finely controlling the fabrication of functional nanoarchitectures.

## 2. Results and Discussion

**Figure 1:**
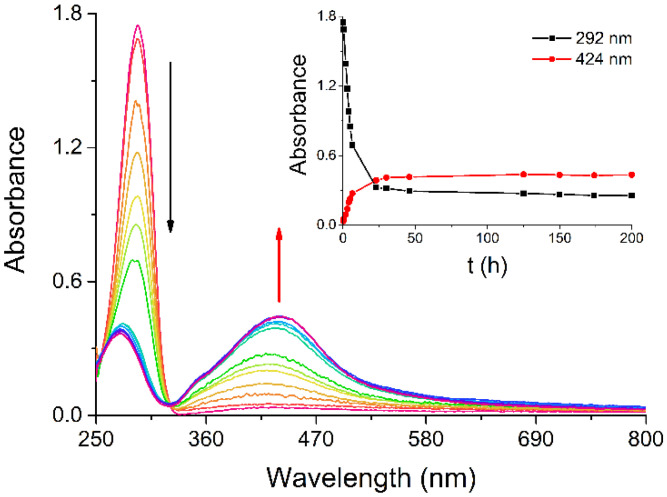
UV/Vis spectral change observed during the kinetics of the spontaneous AgNP formation from Ag(acac). Inset: absorbance of the spectra in the main graph measured at fixed wavelength (292 and 424 nm) vs. time. Experimental conditions: Ag concentration: 14.4 µg/mL; total time: 200 h.

Among the synthetic methods for preparing AgNPs, we chose one that may yield particles with high surface reactivity, such as the protocol devised by Giuffrida et al., which leads to naked AgNPs in water [16]. As reported in the literature, the absorption band observed at 292 nm for a water solution of silver(I) acetylacetonate (Ag(acac)) slowly decreases in intensity while a broad new band forms around 424 nm, in the region typical for AgNPs (Figure 1) [16,17]. The spontaneous decomposition of Ag(acac) has been monitored by UV/Vis spectroscopy to examine the kinetics of AgNP formation at room temperature and determine the time required for their complete synthesis. AgNP nucleation begins within the first minutes, as indicated by the overall spectral change (Figure 1) and the plots of the absorbance at the absorption maxima vs. time (Figure 1, inset), with the reaction being substantially over after about 23 h from Ag(acac) dissolution.

**Figure 2:**
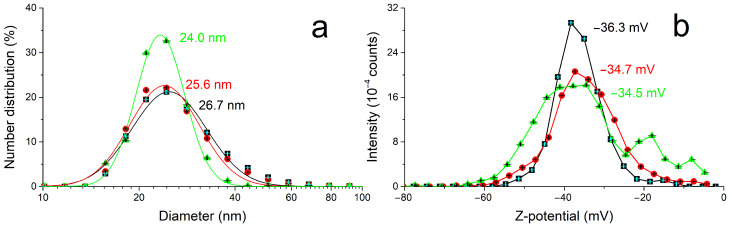
DLS (**a**) and Z-potential (**b**) measurements of an AgNP colloidal solution. Experimental conditions: Ag content: 14.4 µg/mL. Error bars are represented by the black and cyan crosses inside the symbols. Fitting in (**a**): R^2^ = 0.984 (black); 0.984 (red); 0.994 (green).

**Figure 3:**
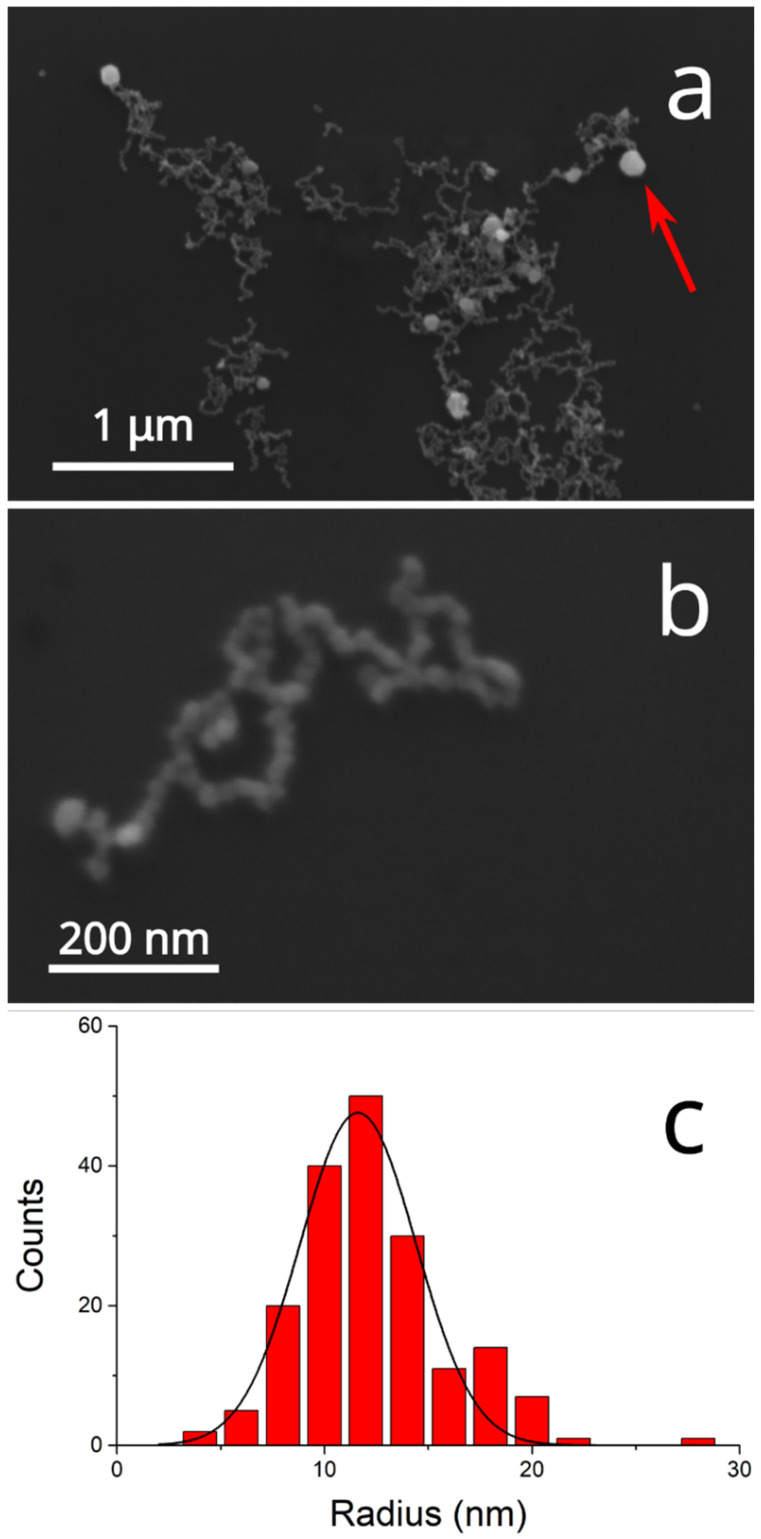
SEM micrographs (**a**,**b**) and empirical size distribution (**c**) of AgNPs formed by the spontaneous decomposition of Ag(acac) in water deposited by drop casting/blotting on a silicon chip. The red arrow in (**a**) indicates one of a few Ag nanoplatelets (see Figure S1 for EDX) that form alongside the nanoparticles. Experimental conditions: Ag concentration: 14.4 µg/mL Fitting in (**c**): mean AgNP radius = (12 ± 3) nm; R^2^ = 0.95.

The size and electrokinetic potential of the AgNPs have been characterized in solution through DLS (Figure 2) and compared to the morphology and composition analyses by SEM and EDX after deposition on a flat silicon substrate, with a very good agreement within the results and with the literature [16,17]. In particular, the mean value of AgNP diameter and Z-potential in solution measured (25 ± 1) nm and (−35 ± 1) mV, respectively. Figure 3 presents the SEM micrographs of the deposited nanomaterial, showing the presence of nanoparticles of (24 ± 6) nm mean diameter aggregated in linear chains accompanied by a few Ag nanoplatelets (red arrow in Figure 3a; EDX in Figure S1a) with diameters in the hundreds of nanometers.

The long-term stability of the AgNP suspensions and the effects of aging are tested by checking the UV/Vis spectrum of a 3 mL aliquot of the colloid (Ag content 14.4 µg/mL) for any sign of precipitation after being left undisturbed for one week at RT and ambient light conditions. The negligible difference between the absorption spectra recorded before and after a gentle homogenization of the aged colloid (Figure S2) indicates that the AgNPs are stable and that no relevant precipitation occurs over this period.

**Figure 4:**
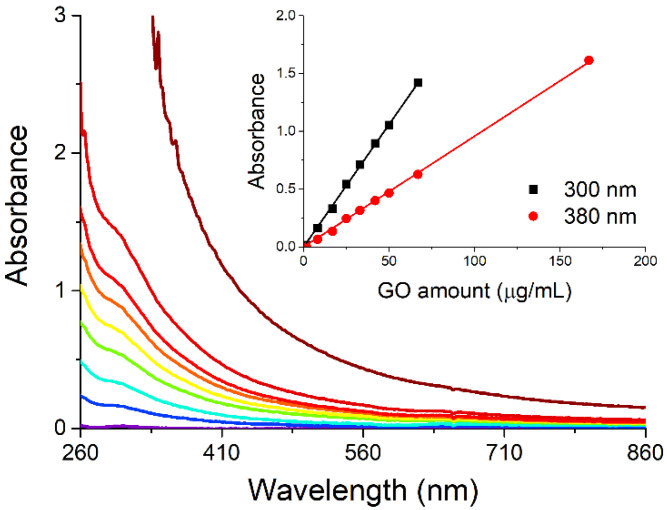
UV/Vis spectra of GO suspensions in UPW as a function of nanosheet concentrations (0–167 µg/mL). Inset: absorbance of the spectra in the main graph measured at fixed wavelength (300 and 380 nm) vs. GO concentration. Linear fit parameters at λ = 300 nm (black): slope: (0.0212 ± 0.0002) mL µg^–1^ cm^–1^; intercept: set to zero; R^2^ = 0.9996. Linear fit parameters at λ = 380 nm (red), slope: (0.00957 ± 0.00008) mL µg^–1^ cm^–1^; intercept: set to zero; R^2^ = 0.9995.

Concerning graphene oxide (GO), we chose to suspend its nanopowders through ultrasounds, a method that allows the control of the level of fragmentation of GO nanosheets [18], by sonicating the solid in water for nine hours in an ice bath. The stability of the GO suspension on increasing the nanosheet concentration has been checked by recording the spectra of samples containing GO between 1.7 and 67 μg/mL (Figure 4). The plot of absorbance at fixed wavelength vs. the amount of GO present in the suspensions (inset in Figure 4) indicates a linear dependence of these two variables, in agreement with Beer’s law. Thus, in the concentration range used in the present study, GO does not suffer aggregation phenomena when its concentration is increased.

**Figure 5:**
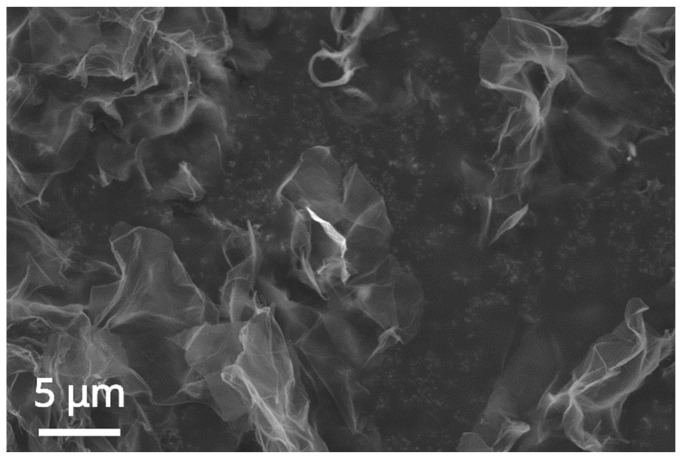
SEM micrograph of the GO nanosheets deposited on a specimen stub. Experimental conditions: freeze-dried GO powder onto a conductive adhesive-coated specimen stub.

As expected for a carbon 2D nanomaterial deposited on a conductive adhesive-covered specimen stub, the single- or few-layer nanosheets appear semitransparent and wrinkled (Figure 5). They are also easily pierced by the electron beam, and the EDX analysis on the nanolayers leads only to detecting the conductive adhesive-coated specimen stub material [19].

The nanocomposite consisting of AgNP-decorated GO nanosheets is synthesized in situ by forming the metal nanoparticles in the presence of the suspended 2D nanomaterial, whose surface can act as a nucleation site, simply adding Ag(acac) to the suspensions having different GO content. The results of this approach are then compared to the ex situ synthesis, where previously synthesized AgNPs are later added to the GO suspensions to highlight any supramolecular interaction between the two components that may contribute to the formation of AgNPs@GO nanocomposites.

**Figure 6:**
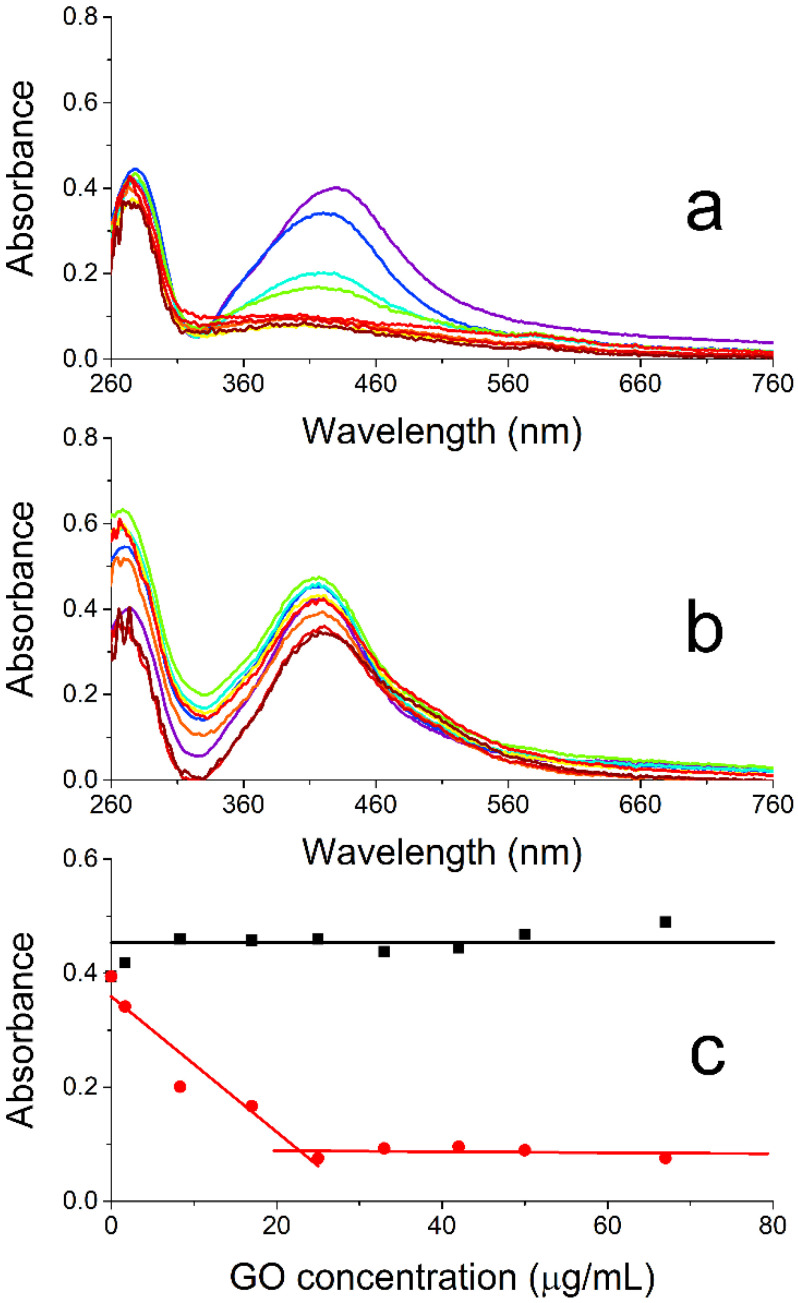
(**a**,**b**) UV/Vis spectra of AgNPs@GO suspensions upon increasing the concentration of GO while keeping that of AgNPs constant, recorded 24 h after mixing the components. Synthetic approach: (**a**) in situ; (**b**) ex situ. (**c**) The absorbance of AgNP@GO nanocomposites in (**a**,**b**) measured at the plasmon band maximum (420 nm) vs. GO concentration for in situ (red; from graph (**a**)) and ex situ (black; from graph (**b**)) protocols; the linear segments act as a guide for the eye. All the spectroscopic data have been corrected for GO absorbance (see Figure S3 for uncorrected data). Experimental conditions: Ag content: 14.4 μg/mL; GO content: 0–67 μg/mL.

Figure 6a presents the UV/vis absorption spectra recorded 24 h after preparing different nanocomposites with an in situ approach by mixing increasing amounts of GO (final concentrations: 0–67 µg/mL) with different aliquots of an Ag(acac) solution right after the dissolution of this salt (final Ag concentration: 14.4 µg/mL). The spectra are subtracted by the absorption contribution of the suspended GO nanosheets (see Figure S3 for uncorrected data). The spectral changes observed for the suspensions on increasing the GO content highlight a substantial effect exerted by the presence of this nanomaterial at the very beginning of Ag(acac) spontaneous decomposition. The initial increase in the number of nanosheets at the beginning of the AgNP formation causes a strong hypochromicity and broadening of the AgNP plasmonic band (Figure 6a and red symbols/lines in Figure 6c) up to 25 μg/mL of GO. A further increase in the GO concentration does not cause any additional relevant change in the plasmon band, which reaches a plateau and nearly merges with the baseline at this point.

As per the ex situ protocol, increasing amounts of GO (final concentrations: 0–67 µg/mL) are added to different aliquots of a preformed silver colloid (final Ag concentration: 14.4 µg/mL). Figure 6b compares the spectra of the resulting suspensions recorded 24 h after mixing the two nanocomponents and subtracted by the GO absorption (see Figure S3 for uncorrected data). These measurements indicate some oscillation across the whole spectrum, especially in the UV region, on increasing the GO content and the formation of a small shoulder at the red edge of the plasmon band (~500 nm). Also, the changes in absorbance measured at the absorption maximum (420 nm; Figure 6c black symbols/lines) underline that not much happens to the AgNPs when increasing GO concentration besides a feasible, slight increase in the nanoparticle tendency to self-aggregate. The absence of relevant interactions between the two nanomaterials in this protocol can be easily explained by both having a negative zeta potential (AgNPs: −35 mV, this work; GO: −33 mV, ref. [20]). The formation of the nanocomposite would, thus, require either a cationic species acting as a mediator (against atom economy) or, as per our in situ approach, simply the presence of the 2D nanomaterial at the beginning of AgNP formation, where the nanosheets could provide nucleation sites for the growth of the metal nanoparticles.

The different behavior highlighted by the comparison between the red and black data in Figure 6c can be interpreted as an increasing engagement of the forming AgNPs in their interaction with the GO nanosheets with the increase in nanosheet content, which implies the parallel increment in the number of available binding sites for the nanoparticles. While even more AgNPs will grow/aggregate on the surface of the nanosheets, fewer NPs will remain free in solution and contribute to the plasmon band centered around 420 nm. If enough GO is present at the beginning of the nanoparticle growth (ca. 25 μg/mL), all the AgNPs will have formed onto the nanosheets, and adding more GO will not affect the optical properties of the suspensions any further, reaching a maximum mass ratio of 0.58 between Ag and GO.

**Figure 7:**
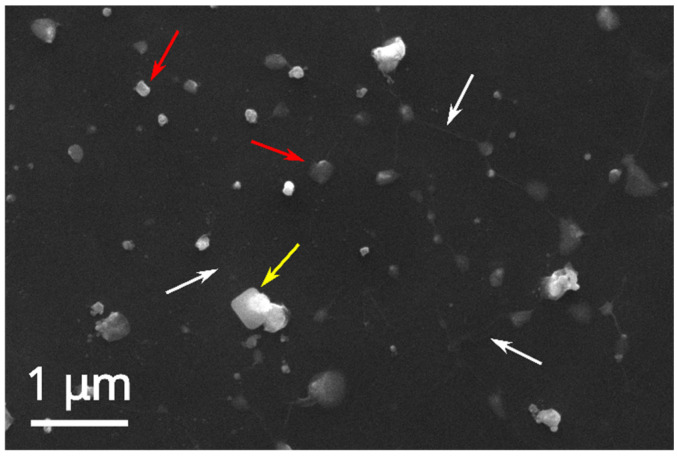
SEM micrograph of AgNPs@GO, formed by an in situ approach, deposited on a silicon chip by drop casting/blotting. The presence of GO nanosheets is made visible by some wrinkles of the semitransparent ultrathin nanosheets (white arrows), while the red arrows point to AgNPs (same EDX profile as in Figure S1a), and the yellow one indicates a NaCl crystal (see EDX in Figure S1b). Experimental conditions: Ag content: 14.4 µg/mL; GO content: 67 µg/mL.

The SEM and EDX analyses of the AgNOs@GO nanocomposite formed by the in situ approach and deposited on silicon chips (Figure 7, same EDX profile as Figure S1) confirm the presence of silver nanoparticles (red arrows) above and below the somewhat wrinkled, ultrathin graphene oxide film (white arrows). These samples present a broad NP size distribution, starting from AgNPs having the same diameter as those formed without GO (~25 nm) through bigger and more commonly found particles of ca. 200 nm diameter, up to 400 nm NP clusters.

The stability of the nanocomposite suspensions having the maximum Ag/GO mass ratio (0.58) has been monitored by spectroscopy over three weeks, and no relevant changes are observed in the spectrum profile or absorbance values (Figure S4). This evidence points to the stability of the nanocomposite over time, which is a fundamental prerequisite for its processability when any practical application is to be attained.

## 3. Materials and Methods

Materials: Silver(I) acetylacetonate (Ag(acac)) and ultrapure water (UPW) were purchased at VWR (Milan, Italy); graphene oxide nanopowder (GO) was purchased at Abalonix (Oslo, Norway). All the chemicals were used without any further purification.

AgNP synthesis: AgNPs were obtained in a single step by the spontaneous decomposition of Ag(acac) at RT without external reducing agents and stabilizing ligands [16]. In total, 5 mg of Ag(acac) was dissolved in 120 mL of UPW, and the solution was left undisturbed for 24 h at RT and ambient light. Since no precipitate was detected at the end of the reaction, the final colloid concentration expressed in Ag atoms was 0.2 mM or 22 µg/mL. Once formed, the Ag colloid was stable for over six months.

GO suspensions in UPW: UPW suspensions of GO were prepared from the dry powders, dispersing them through sonication [18] to reach a final GO concentration of 0.5 mg/mL. A good dispersion of the GO flakes was obtained by sonicating the suspensions over 9 h (final pH~7).

AgNPs@GO ex situ synthesis: The preformed Ag colloid was mixed with various aliquots of the GO suspension to attain samples with increasing GO concentrations (Table 1) while keeping the concentration of the AgNPs constant (Ag atom content: 0.133 mM or 14.4 µg/mL).

AgNPs@GO in situ synthesis: The Ag(acac) solution was mixed with GO suspension immediately after dissolving the acetylacetonate salt in UPW. As in the case of ex situ synthesis, the samples were prepared with increasing GO concentrations (Table 1) while keeping the concentration of the AgNPs constant (Ag atom content: 0.133 mM or 14.4 µg/mL).

**Figure 8:**
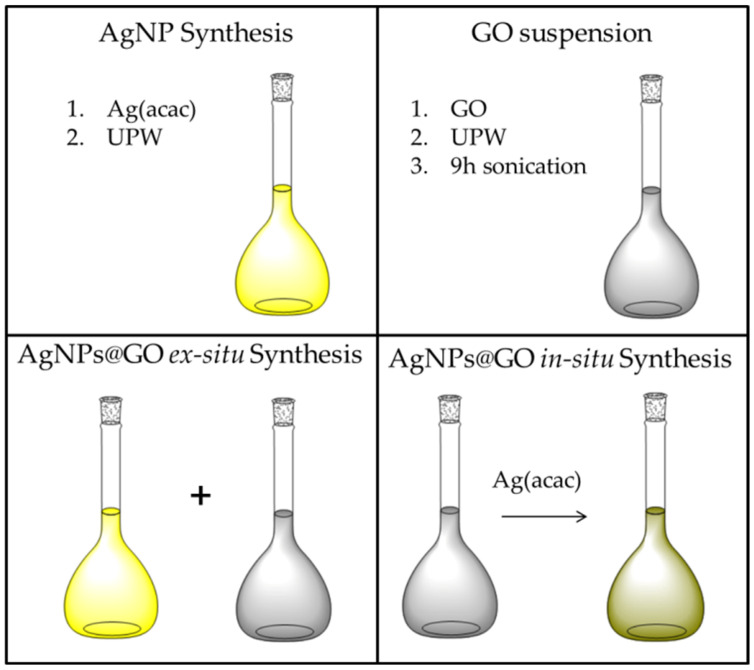
Schematic representation of AgNP synthesis; GO suspension preparation; AgNPs@GO ex situ and in situ synthesis.

A schematic representation of the above-described procedures is shown in Figure 8.

Characterizations: UV/Vis absorption spectra of all the suspensions were measured in 1 cm PMMA cuvettes with a Stellarnet BLACK-Comet-SR diode array spectrophotometer (StellarNet Inc., Tampa, FL, USA) equipped with a combined deuterium/tungsten halogen lamp (Stellarnet mod. SL5) and a multimodal fiber optic cable (length: 1 m; diameter: 1 mm; optical range: 190–1100 nm). The samples were gently inverted once to homogenize them before recording the spectra.

The mean particle size and electrokinetic potential of the AgNPs in solution were determined by the DLS and zeta potential techniques at 25 °C using a Zetasizer Nano ZS instrument (Malvern Panalytical, Malvern, UK) equipped with a helium-neon 4 mW laser (λ = 632.8 nm) and at a scattering angle of 173°. The values for the radium and electrokinetic potential of the AgNPs were obtained as a mean over three measurements, with the reported uncertainty being the average absolute deviation.

AgNP and AgNPs@GO samples for the SEM-EDX analyses were prepared by drop casting/blotting the suspensions on flat silicon substrates, whereas the GO nanosheets were deposited as a freeze-dried powder onto a conductive adhesive-coated specimen stub. The images were recorded using an FEI Quanta FEG 450 instrument (Hillsboro, OR, USA) operating with an accelerating voltage of 10 kV under high vacuum conditions (10^−4^–10^−5^ Pa). A semi-quantitative chemical analysis was carried out using energy-dispersive X-ray spectroscopy (EDAX, Ametek, Tokyo, Japan). The SEM micrographs were analyzed in Fiji ver. 1.54f [21] using the ComDet plugin ver. 0.5.5 [22], and a mean value for the AgNP radius was roughly estimated by fitting the empirical frequency distribution (counts vs. radius) to a normal distribution.

## 4. Conclusions

In this study, we developed a facile, one-pot, and green synthetic method to create Ag nanoparticle-decorated graphene oxide (AgNPs@GO) nanocomposites. We exploited the spontaneous decomposition of silver(I) acetylacetonate in water, a green method known to yield naked AgNPs [16], in the presence of GO nanoflakes to produce the nanocomposite in one reaction step. This in situ method was then compared to an ex situ approach, where previously synthesized AgNPs were added to GO nanosheets, the latter showing no indication of any interaction between the two nanomaterials in solution.

AgNPs and their GO nanocomposites were characterized using various techniques, such as UV/Vis absorption spectroscopy, DLS, zeta potential, and SEM-EDX, observing good long-term stability for the nanohybrids. Our approach may open the way where the properties of GO, such as its surface absorption or structural strengthening capabilities, and those of silver nanoparticles, e.g., their plasmonic or antibacterial properties, can work together toward more complex functional architectures. Indeed, our nanocomposites will soon be employed aiming at toughening and antibacterial nano-additives in implantable bone substitutes and as biosensing nanoplatforms that exploit Surface-Enhanced Raman Scattering signals.

## Figures and Tables

**Table 1:** Ag and GO concentrations in A-I samples.

Sample	Ag Content (µg/mL)	GO Content (μg/mL)
A	14.4	0
B		1.7
C		8.3
D		17
E		25
F		33
G		42
H		50
I		67

## Data Availability

Data is contained within the article and Supplementary Materials.

## Supplementary Materials

The following supporting information can be downloaded at: https://www.mdpi.com/article/10.3390/ijms26020713/s1.
