# Supplementary material for: Easy One-Pot Decoration of Graphene Oxide Nanosheets by Green Silver Nanoparticles
Source: Int J Mol Sci. 2025 Jan 16;26(2):713. doi: 10.3390/ijms26020713 (PMC11766102; doi:10.3390/ijms26020713)
Supplement: Supplementary file 1 [file ijms-26-00713-s001.zip › ijms-3399796-supplementary.pdf]

SUPPORTING INFORMATION

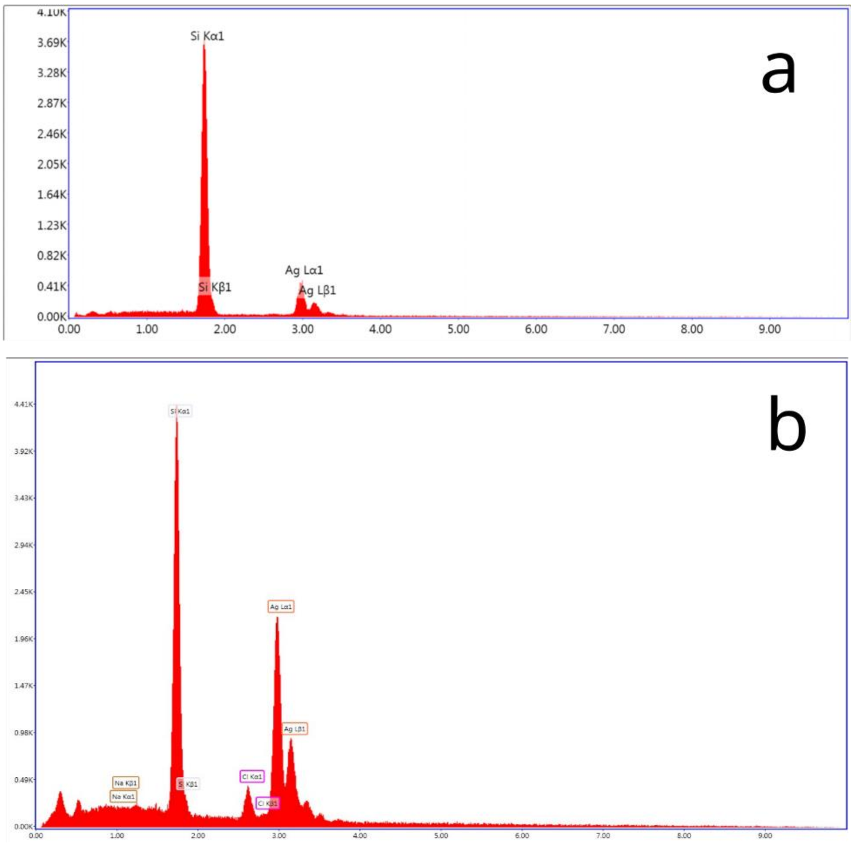

Figure S1. a) EDX spectra of the Ag nanoplatelet in Figure 3a, also representative of the spectrum of AgNP. b) EDX spectrum of the squared crystal in Figure 7 (yellow arrow).

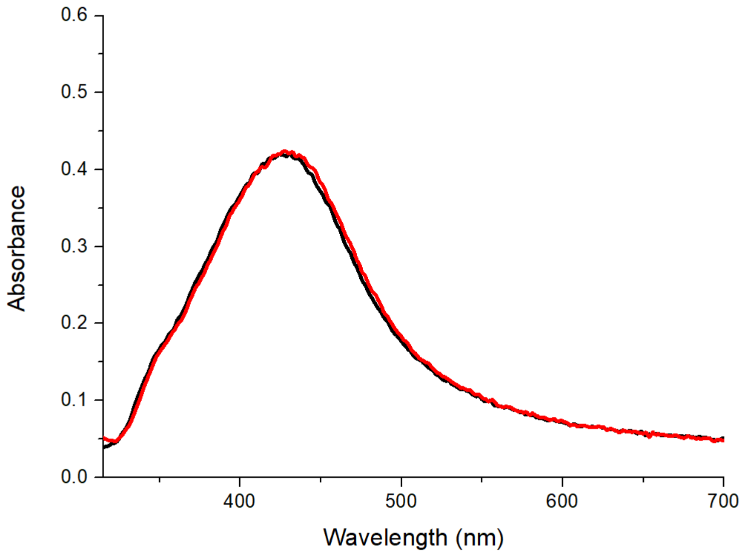

Figure S2. UV/Vis spectra of an aged AgNPs colloidal solution recorded before and after gentle homogenization (black and red curves, respectively). Experimental conditions: aging time: one week; aliquot volume: 3 mL; Ag content: 14.4 µg/mL.

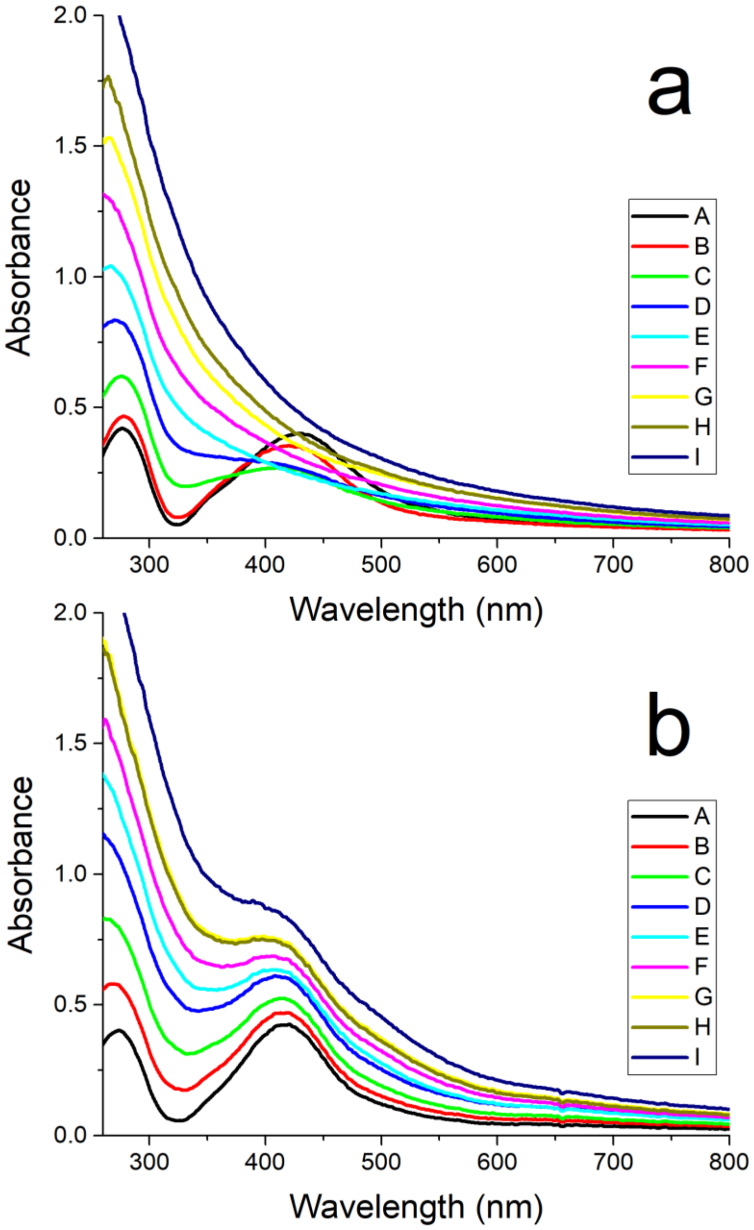

Figure S3. UV/Vis spectra of AgNPs@GO suspensions upon increasing the concentration of GO while keeping that of AgNPs constant, recorded 24 hours after mixing the components. Synthetic approach: (a) *in situ*; (b) *ex situ*. The spectra have <u>not</u> been corrected by the absorbance of GO (see Fig. 4a,b for corrected data. Experimental conditions: Ag content: 14.4 µg/mL; GO content: 0-67 µg/mL.

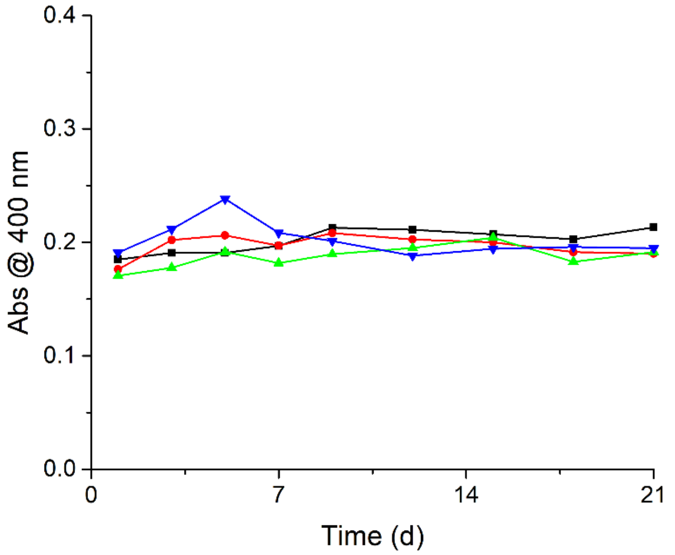

Figure S4. Absorbance of the AgNPs@GO suspensions (four replicas) monitored at a fixed wavelength (400 nm) over three weeks. Experimental: Ag content: 14.4 µg/mL; GO content: 25 µg/mL; Ag/GO ratio: 0.58.
